# Construction of S-Scheme Heterojunction MoS_2_/TiO_2_ and Investigation of the Photocatalytic Inhibition Performance

**DOI:** 10.3390/ijms27219763

**Published:** 2026-10-30

**Authors:** Qiqin Lu, Guijun Yang, Ting Han, Hui Nan

**Affiliations:** 1 School of Chemical Engineering, Qinghai University, Xining 810016, China; 2 School of Mechanical Engineering, Qinghai University, Xining 810016, China

**Keywords:** anodic oxidation, MoS_2_, antibacterial activity, S-scheme heterojunction

## Abstract

TiO_2_ possesses excellent photocatalytic antibacterial performance. Owing to its unique structural and physicochemical characteristics, TiO_2_ has been extensively applied in energy, environmental remediation, and biomedical fields. Herein, a hierarchical one-dimensional (1D) TiO_2_ architecture consisting of a well-distributed nanotube bottom layer whose surface nanowires interconnect into groove structures was fabricated on titanium foil via anodic oxidation, and molybdenum disulfide (MoS_2_) nanosheets were further grown in situ on the TiO_2_ surface to construct MoS_2_/TiO_2_ composite photocatalysts. The as-prepared composites exhibit superior hydrophilicity and prominent photocatalytic activity under full-spectrum simulated-solar irradiation, achieving a bactericidal efficiency of 86% against *Staphylococcus aureus*. Characterization results confirm that MoS_2_ nanoflower-like spheres are uniformly loaded on the surface of the 1D TiO_2_. An intimate S-scheme heterojunction is successfully fabricated between the two components, which significantly promotes the migration and separation of photogenerated charge carriers. Electron paramagnetic resonance (EPR) tests demonstrate that superoxide radicals (•O_2_^−^) and hydroxyl radicals (•OH) are the dominant active species responsible for the efficient antibacterial behavior. This work reveals the significant application potential of MoS_2_/TiO_2_ composite photocatalysts and provides a feasible theoretical basis for the practical development and application of high-efficiency photocatalytic antibacterial materials.

## 1. Introduction

Industrial wastewater discharged from industrial production contains abundant hazardous contaminants, including organic dyes, toxic chemicals, and pathogenic microorganisms, which pose serious threats to the ecological balance, human health, and the survival of aquatic organisms [1,2,3,4,5]. In addition, bacterial infections occurring during or after surgery have long remained a major challenge in clinical settings [6,7]. Photocatalysis is an environmentally friendly technology that converts solar energy into usable chemical energy without generating secondary pollution, offering distinct advantages and continually attracting extensive research interest [8,9,10]. Among various photocatalytic materials, TiO_2_-based photocatalysts are universally recognized as ideal candidates because of their non-toxicity, low cost, rapid degradation rate, mild reaction conditions, and excellent cycling stability [11,12,13,14]. However, the inherent wide band gap and low separation efficiency of photogenerated charge carriers severely limit the large-scale practical application of pristine TiO_2_.

Constructing heterojunctions by coupling TiO_2_ with narrow-bandgap visible-light-driven semiconductors (e.g., MoS_2_, CdS, NiS) has been proven to be an effective strategy to optimize the optical absorption and carrier-separation performance of TiO_2_ [15,16]. As a typical two-dimensional layered material, MoS_2_ features a suitable narrow band gap and excellent electrical conductivity, and these intrinsic properties render it a promising modifier for boosting the photocatalytic performance of metal oxide semiconductors [17,18,19]. Sun et al. [20] successfully fabricated MoS_2_@TiO_2_ composite nanostructured arrays through a facile approach, in which MoS_2_ nanosheets were uniformly loaded on anodized TiO_2_ nanotube arrays; the optimized composite achieved a methylene blue photodegradation efficiency of 92.10%. Li et al. [21] verified via sacrificial agent experiments that MoS_2_/TiO_2_ composites prepared by a two-step solvothermal method form a Type-I heterojunction, which remarkably enhances the photocatalytic degradation performance toward Rhodamine B (RhB). Moreover, Liu et al. [22] fabricated a novel BC/MoS_2_/TiO_2_ composite with the dual functions of wastewater purification and antibacterial activity. Despite substantial progress in optimizing the performance of MoS_2_/TiO_2_ composites, most existing studies primarily focus on conventional heterojunction systems and pollutant-degradation applications. In particular, the construction and photocatalytic antibacterial mechanism of S-scheme MoS_2_/TiO_2_ heterojunctions, which possess superior redox capability compared with traditional heterojunctions, remain insufficiently explored. Clarifying the underlying S-scheme charge-transfer and antibacterial mechanism is therefore of great significance for guiding the design and preparation of high-efficiency multifunctional photocatalysts.

To date, most reported S-scheme MoS_2_/TiO_2_ heterostructures have been predominantly exploited for dye degradation and solar-driven hydrogen evolution, while their applications in microbial disinfection remain underexplored [23]. Moreover, previously developed S-scheme antibacterial photocatalysts rely on distinct material systems, including conjugated-polymer/TiO_2_(B) heterojunctions [24], g-C_3_N_4_-based composite photocatalysts [25], and CuS/Bi_5_O_7_I hybrids [26], which differ substantially from the purely inorganic, dual-component MoS_2_/TiO_2_ system investigated herein. Further literature survey indicates that most existing MoS_2_/TiO_2_ antibacterial studies adopt Type-II or Z-scheme charge transfer pathways [27,28], as well as nanozyme-photothermal synergistic catalytic strategies [22,29]. Notably, systematic exploration of the S-scheme antibacterial mechanism is still scarce, especially for self-supported one-dimensional (1D) MoS_2_/TiO_2_ architectures. Such unique structural configuration enables directional charge transport and simultaneously maintains powerful reductive (•O_2_^−^) and oxidative (•OH) active sites. To the best of our knowledge, the integration of MoS_2_/TiO_2_ antibacterial performance evaluation and in-depth mechanistic clarification has rarely been realized in prior research.

In this work, a high-efficiency MoS_2_/TiO_2_ heterojunction photocatalyst was successfully fabricated for enhanced photocatalytic antibacterial performance. Specifically, a hierarchical one-dimensional (1D) TiO_2_ nanotube–nanowire architecture was prepared via two-step anodic oxidation on titanium foil, and MoS_2_ nanoflowers were subsequently grown in situ on the TiO_2_ surface, forming an intimate interfacial heterojunction. The photocatalytic antibacterial performance of the optimized composite was evaluated using *Staphylococcus aureus* as the model bacterium, delivering a bactericidal efficiency of 86% under full-spectrum simulated-solar illumination. Furthermore, based on band-structure analysis, an S-scheme photocatalytic charge-transfer mechanism was proposed to elucidate the enhanced activity of the MoS_2_/TiO_2_ composite, with superoxide (•O_2_^−^) and hydroxyl (•OH) radicals confirmed as the dominant reactive species by EPR. This work provides a fundamental theoretical basis for the further exploration and performance optimization of TiO_2_-based antibacterial photocatalysts, and offers a mechanism-resolved, practically deployable route toward high-efficiency antimicrobial surfaces.

## 2. Results and Discussion

### 2.1. Characterization of Photocatalyst

**Figure 1:**
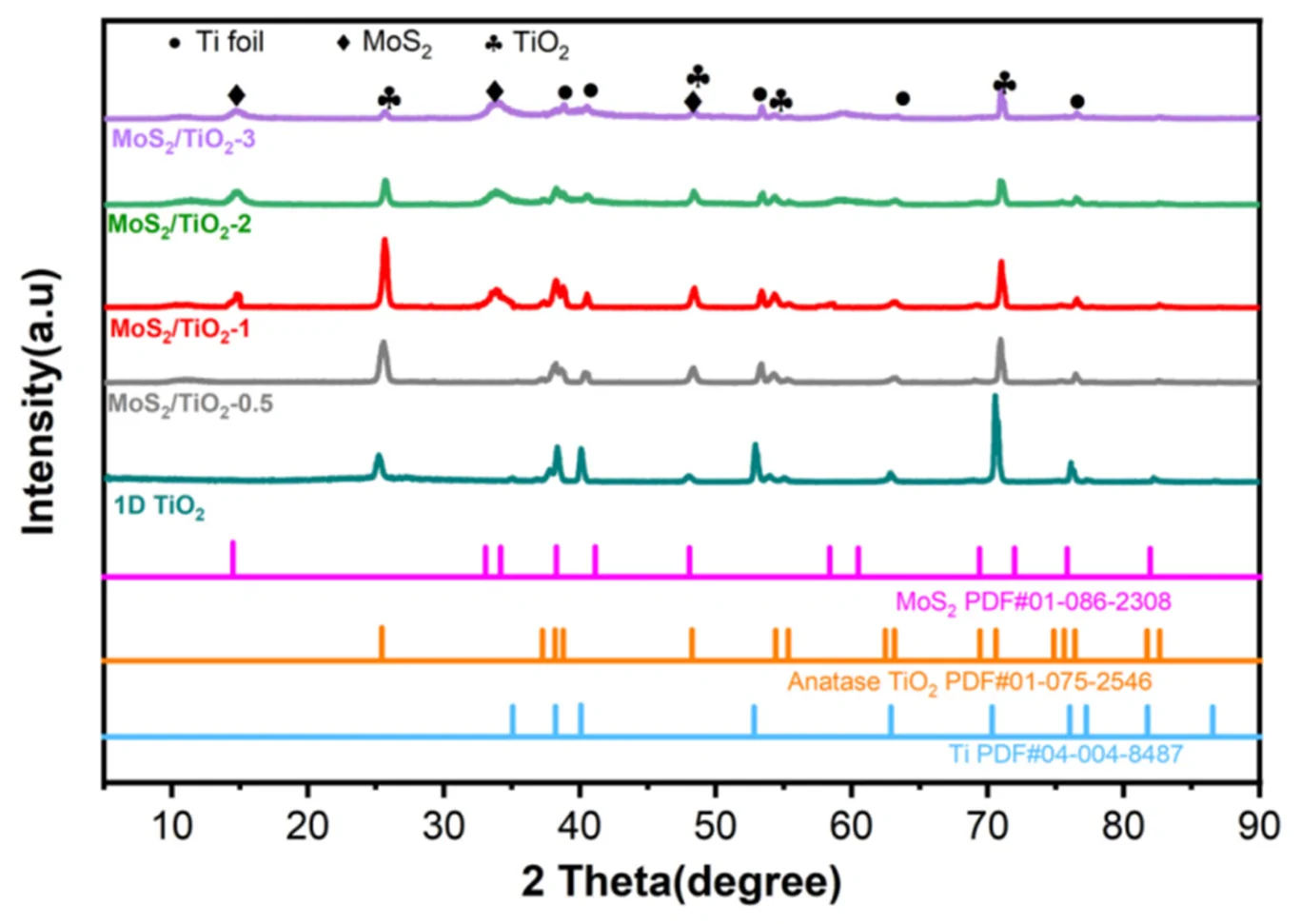
XRD patterns of TiO_2_ and MoS_2_/TiO_2_ composite materials.

The structures of pristine 1D TiO_2_ and MoS_2_/TiO_2_ composites were characterized via X-ray diffraction (XRD) (Figure 1). The diffraction peaks located at 25.56°, 48.19°, and 54.27° are well indexed to the anatase phase of TiO_2_ [30]. No impurity peaks are observed, demonstrating the high phase purity of the as-prepared TiO_2_. Characteristic diffraction peaks assigned to the (002) plane of MoS_2_ at 14.485° and the (110) plane at 58.357° are also clearly detected [31]. Moreover, these MoS_2_ peaks become progressively sharper with increasing MoS_2_ loading, further verifying the successful fabrication of the MoS_2_/TiO_2_ composite materials.

**Figure 2:**
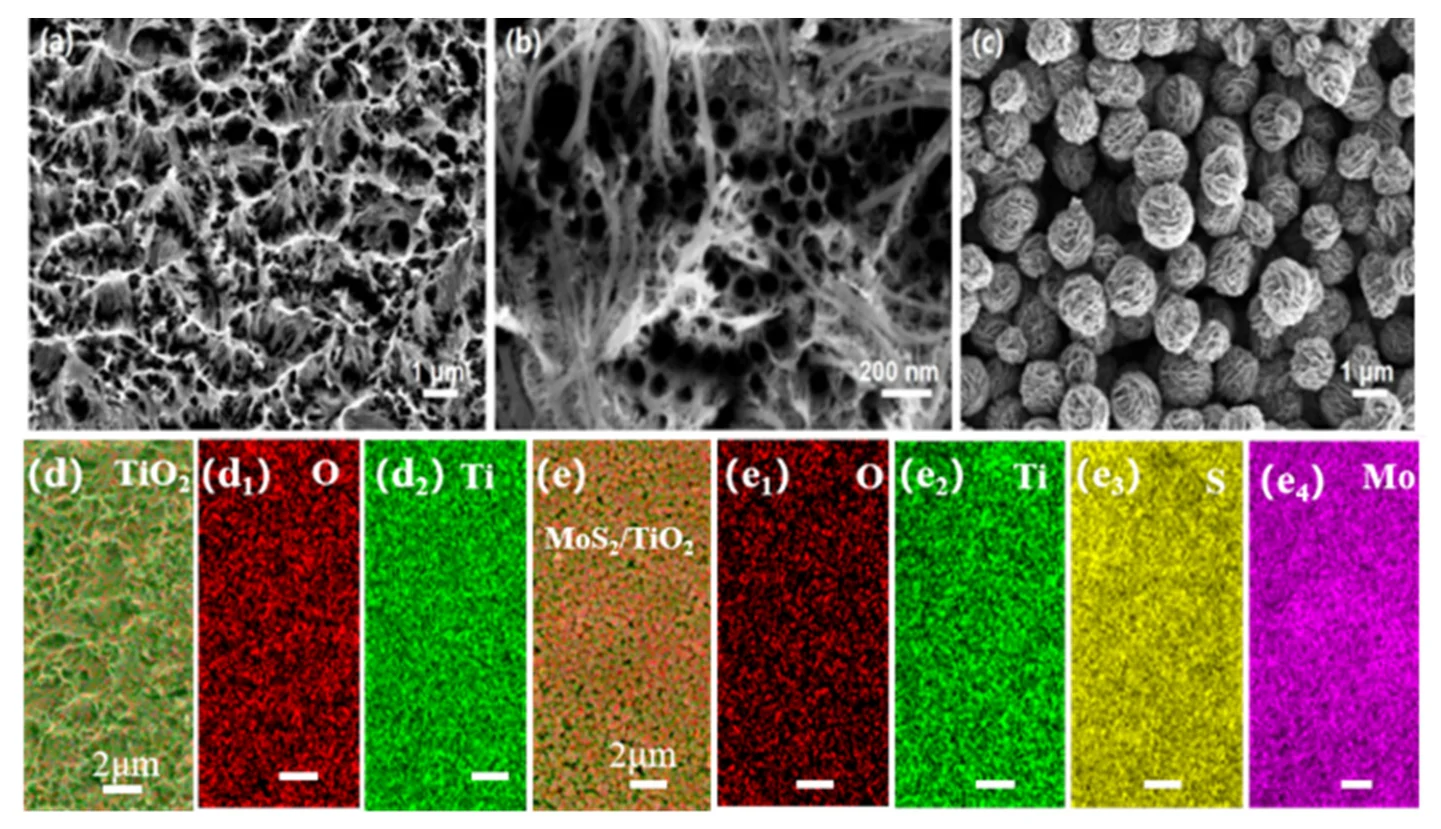
SEM images and elemental analysis of 1D TiO_2_ and MoS_2_/TiO_2_: (**a**) low-magnification SEM image of the surface of the pristine TiO_2_-covered titanium foil; (**b**) high-magnification SEM image revealing the bilayer TiO_2_ nanotube–nanowire architecture; (**c**) SEM image of the MoS_2_/TiO_2_ composite showing MoS_2_ nanoflower spheres anchored on the TiO_2_ surface; (**d**) EDS elemental mapping of pure TiO_2_: (**d_1_**) O, (**d_2_**) Ti; (**e**) EDS elemental mapping of the MoS_2_/TiO_2_ composite: (**e_1_**) O, (**e_2_**) Ti, (**e_3_**) S, (**e_4_**) Mo.

Scanning electron microscopy (SEM) combined with energy-dispersive X-ray spectroscopy (EDS) was employed to characterize the morphology and elemental composition of pristine 1D TiO_2_ and MoS_2_/TiO_2_ composites. The low-magnification view shows that the surface of the titanium foil is uniformly covered with hill-like TiO_2_ structures (Figure 2a). As observed from the high-magnification image in Figure 2b, TiO_2_ grows as a bilayer structure on the titanium foil. The bottom layer consists of well-distributed TiO_2_ nanotubes, while the tips of nanowires extending from the nanotube surfaces interconnect to form groove structures. This 1D TiO_2_ architecture, assembled from these nanotubes and nanowires, possesses abundant micro- and nanoscale pores. Clearly, MoS_2_ nanosheet-assembled nanoflower spheres are uniformly anchored on the TiO_2_ surface, with abundant interlayer pores between the MoS_2_ nanosheets (Figure 2c).

To further confirm the successful incorporation of MoS_2_ onto TiO_2_, the elemental composition and spatial distribution of the composites were examined by EDS. The elemental mapping clearly reveals that only Ti and O elements are detectable in pristine 1D TiO_2_. In contrast, characteristic Mo and S signals were observed for the MoS_2_/TiO_2_ composite, and these two elements were homogeneously distributed across the material surface (Figure 2d,e). This result confirms the successful loading of MoS_2_ onto the 1D TiO_2_ substrate, consistent with the XRD analysis.

**Figure 3:**
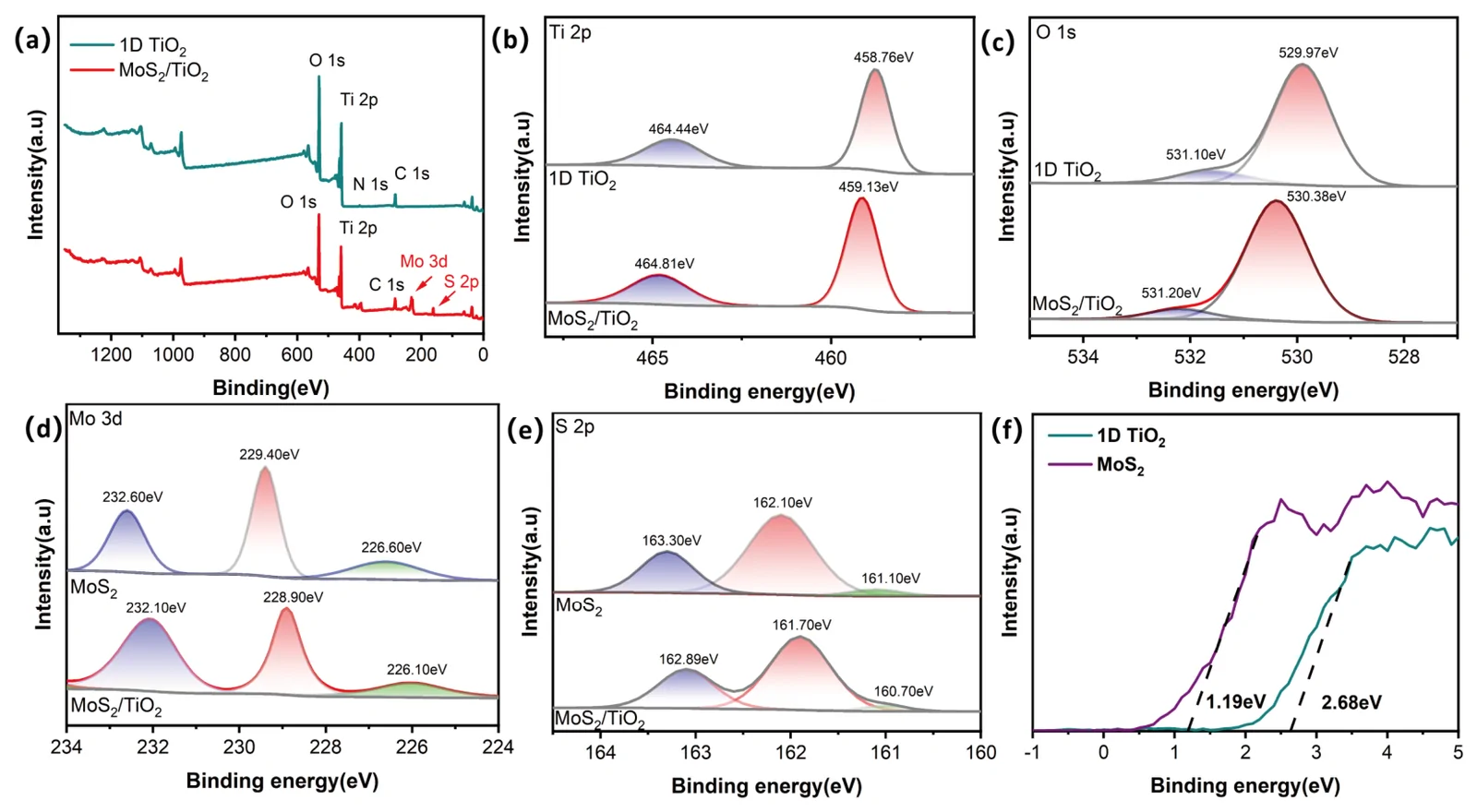
XPS of 1D TiO_2_ and MoS_2_/TiO_2_ (**a**), Ti 2p (**b**), O 1s (**c**), Mo 3d (**d**), S 2p (**e**), the XPS-VB of 1D TiO_2_ and MoS_2_ (**f**).

X-ray photoelectron spectroscopy (XPS) was employed to analyze the surface elemental composition and chemical valence states of pristine 1D TiO_2_, pristine MoS_2_, and MoS_2_/TiO_2_ composites. As can be seen from the survey spectra of 1D TiO_2_ and MoS_2_/TiO_2_ (Figure 3a), the pristine 1D TiO_2_ sample is composed mainly of Ti and O elements, together with a weak N 1s signal (~400 eV) attributable to trace nitrogen introduced by the NH_4_F-based anodization electrolyte, in addition to the ubiquitous C 1s peak arising from surface carbon contamination during XPS measurement. By contrast, the MoS_2_/TiO_2_ composite displays four characteristic elements (Ti, O, Mo, and S), while the weak N 1s feature is no longer resolved after MoS_2_ loading. No impurity phases or by-products are detected, further confirming the successful fabrication of the MoS_2_/TiO_2_ composite.

The two distinct peaks located at 458.8 eV and 464.4 eV were assigned to Ti 2p_3_/_2_ and Ti 2p_1_/_2_ of 1D TiO_2_ (Figure 3b), respectively, confirming that titanium exists mainly in the tetravalent Ti^4+^ state in TiO_2_ [32]. For the MoS_2_/TiO_2_ composite, the Ti 2p_3_/_2_ and Ti 2p_1_/_2_ signals appeared at 459.1 eV and 464.8 eV. The introduction of MoS_2_ exerted no obvious influence on the chemical valence of TiO_2_, and Ti still remained in the Ti^4+^ oxidation state in the composite. Notably, compared with pure TiO_2_, the Ti 2p binding energies of MoS_2_/TiO_2_ shifted toward higher values, demonstrating electron loss of TiO_2_ after coupling with MoS_2_ [33,34].

The TiO_2_ and MoS_2_/TiO_2_ composites showed obvious characteristic O 1s peaks (Figure 3c). In pure TiO_2_, the deconvoluted peak at 529.9 eV belonged to lattice oxygen, while the peak at 531.1 eV was ascribed to surface adsorbed hydroxyl oxygen [35]. Similarly, the O 1s binding energies of the MoS_2_/TiO_2_ composite shifted to higher values relative to 1D TiO_2_, indicating that electron transfer occurs at the heterogeneous interface and that TiO_2_ tends to lose electrons after MoS_2_ immobilization on 1D TiO_2_.

The MoS_2_ and MoS_2_/TiO_2_ composites showed obvious characteristic Mo 3d peaks (Figure 3d,e). The Mo 3d spectrum exhibited two characteristic peaks at 229.4 eV and 232.6 eV, assigned to Mo 3d_5_/_2_ and Mo 3d_3_/_2_, respectively [36]. The S 2s peak located at 226.6 eV corresponds to the sulfide S^2−^ species. For the S 2p spectrum, two distinct peaks at 162.1 eV and 163.3 eV are attributed to S 2p_3_/_2_ and S 2p_1_/_2_ [37]. Notably, the characteristic peaks of Mo 3d and S 2p in the MoS_2_/TiO_2_ heterostructure shift toward lower binding energies. This variation originates from the efficient electron migration from TiO_2_ to MoS_2_ during heterostructure construction, which further induces the formation of an interfacial electric field oriented from TiO_2_ to MoS_2_ at the contact interface [38].

Meanwhile, the XPS valence band spectra of pure TiO_2_ and pristine MoS_2_ were collected (Figure 3f), which can be used to determine the energy difference between the valence band maximum (VBM) and the Fermi level (*E*_f_) [39]. As shown in (Figure 3f), plotted on the E−*E*_f_ scale with the Fermi level set to 0 eV, the valence band onsets are located 2.68 eV and 1.19 eV below the Fermi level for 1D TiO_2_ and MoS_2_, respectively, the Fermi level lies 2.68 eV (TiO_2_) and 1.19 eV (MoS_2_) above the VBM. Combined with the band positions reported on the NHE scale (Table 1), these values yield Fermi level positions of +0.09 V (TiO_2_) and +0.15 V (MoS_2_) versus NHE.

**Figure 4:**
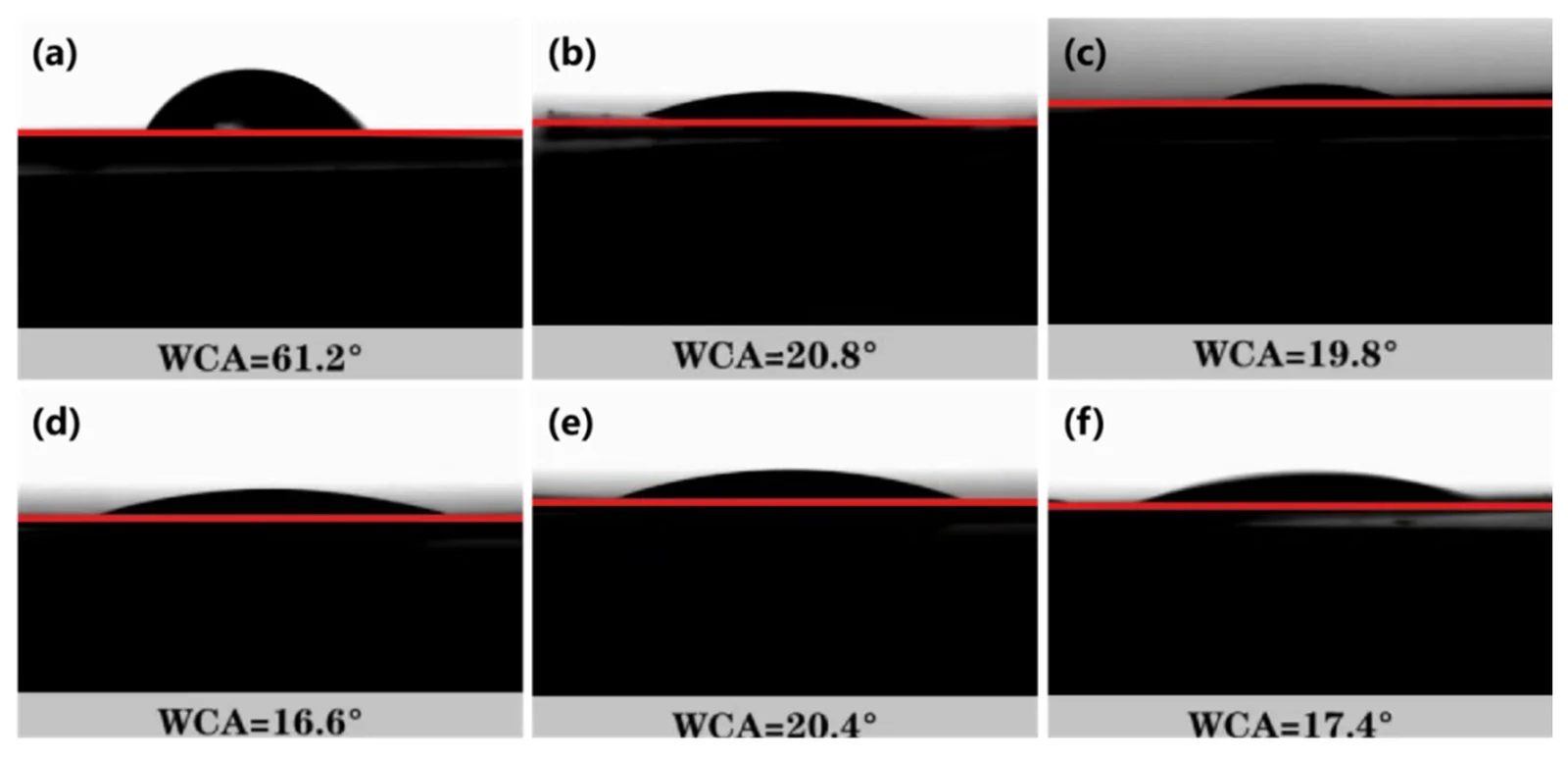
Water contact angles of titanium foil (**a**); 1D TiO_2_ (**b**); MoS_2_/TiO_2_-0.5 (**c**); MoS_2_/TiO_2_-1 (**d**); MoS_2_/TiO_2_-2 (**e**); MoS_2_/TiO_2_-3 (**f**). The red line represents the baseline of the solid substrate surface for contact angle calculation.

The hydrophilicity of MoS_2_/TiO_2_ is an important factor affecting the photocatalytic performance. The hydrophilic properties of the as-prepared samples were evaluated via water contact angle (WCA) measurement under ambient conditions (Figure 4). Compared with pristine titanium foil, the 1D TiO_2_-modified titanium foil exhibited remarkably improved hydrophilicity, with the WCA decreasing from 61.2° to 20.8°. After further MoS_2_ loading, the WCA was reduced even more, confirming that the introduction of MoS_2_ effectively enhances the surface hydrophilicity. Among all samples, the MoS_2_/TiO_2_-1 composite exhibited the minimum WCA value, revealing its superior hydrophilic performance.

It is widely acknowledged that the photocatalytic antibacterial mechanism is primarily dependent on the light-induced generation of reactive oxygen species (ROS). A highly hydrophilic surface facilitates the production and diffusion of ROS while also suppressing bacterial adhesion to a certain extent [40].

### 2.2. Evaluation of Photocatalytic Activity

**Figure 5:**
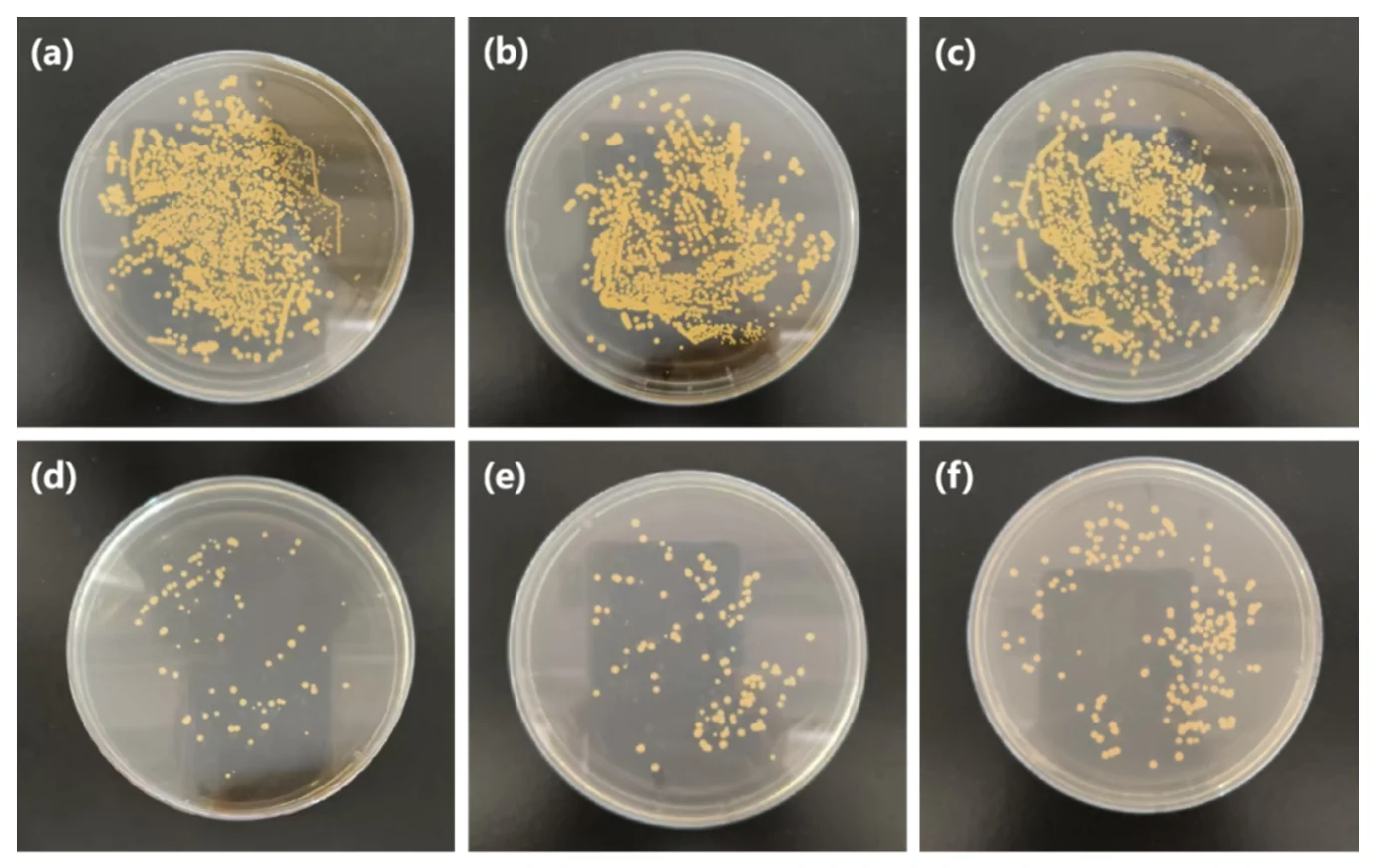
Images of *Staphylococcus aureus* colonies on agar plates: Ti foil (**a**); 1D TiO_2_ (**b**); MoS_2_/TiO_2_-0.5 (**c**); MoS_2_/TiO_2_-1 (**d**); MoS_2_/TiO_2_-2 (**e**); MoS_2_/TiO_2_-3 (**f**).

**Figure 6:**
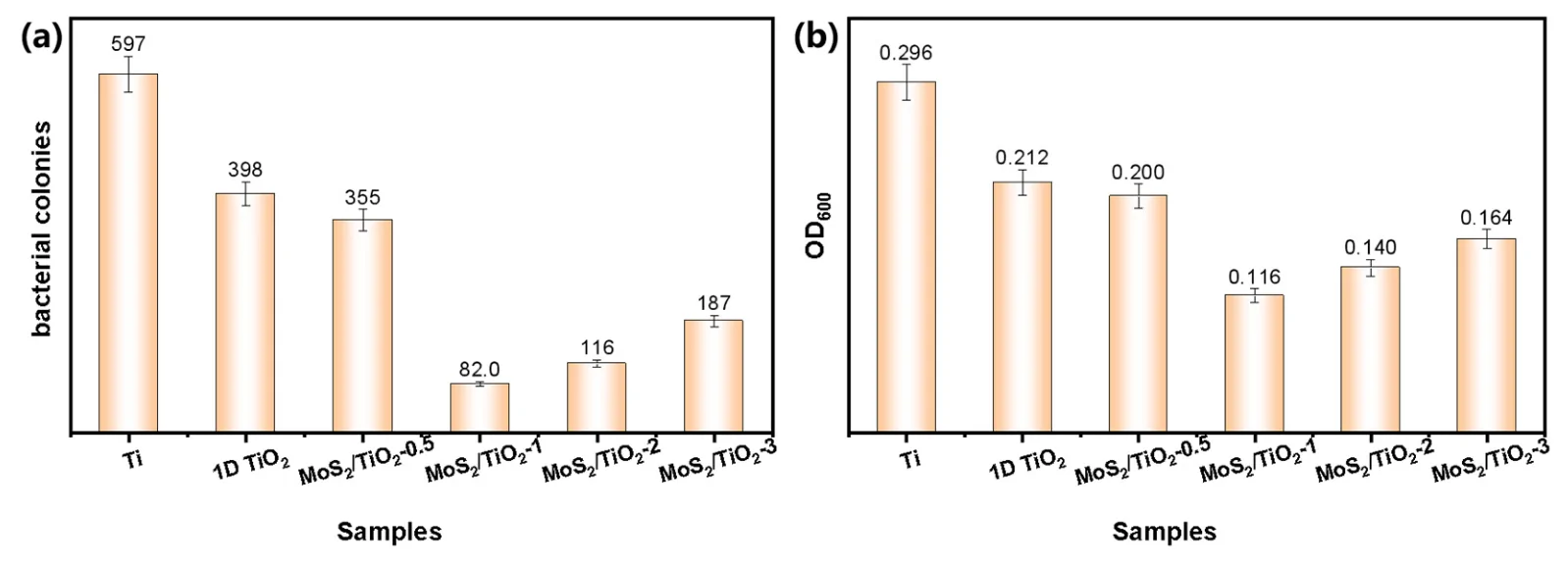
The *Staphylococcus aureus* colony number (**a**) and OD value (**b**) of Ti foil, 1D TiO_2_, MoS_2_/TiO_2_-0.5, MoS_2_/TiO_2_-1, MoS_2_/TiO_2_-2, and MoS_2_/TiO_2_-3.

To investigate the practical applicability of MoS_2_/TiO_2_ composites, photocatalytic antibacterial experiments were conducted. Using *Staphylococcus aureus* as a model bacterium, the photocatalytic antibacterial performance of MoS_2_/TiO_2_ was systematically assessed (Figure 5). First, the bactericidal effect of MoS_2_/TiO_2_ against *Staphylococcus aureus* was evaluated under dark conditions, in which MoS_2_/TiO_2_ exhibited no obvious inhibitory effect on the bacterium in the dark, revealing favorable biocompatibility (Figure S1). Compared with pristine titanium foil (used as the blank control), 1D TiO_2_ exhibited moderate antibacterial activity. After loading MoS_2_, the antibacterial activity of the MoS_2_/TiO_2_ composites was significantly enhanced. According to the statistical results of three parallel experiments, the MoS_2_/TiO_2_-1 composite showed the optimal antibacterial activity, with only approximately 82 colonies observed (Figure 6a). Compared with pure titanium, the MoS_2_/TiO_2_ composite achieved an antibacterial rate of 86% against *Staphylococcus aureus*. Notably, the antibacterial activity first increased and then decreased with increasing MoS_2_ loading content.

This trend is closely associated with the surface hydrophilicity and ROS generation capacity of the composites, consistent with the previous WCA analysis results. An appropriate amount of MoS_2_ loading can further improve the surface hydrophilicity of the composite, thereby facilitating the photogeneration and diffusion of ROS and enhancing the antibacterial effect; however, excessive MoS_2_ loading may cover the active sites of TiO_2_, hinder light absorption and ROS generation, and consequently lead to a decrease in antibacterial activity [40].

To further evaluate the antibacterial inhibition performance, the optical density (OD_600_) value of the bacterial suspension was measured (Figure 6b). Generally, a smaller OD value indicates a lower bacterial concentration, corresponding to fewer viable microorganisms and superior antibacterial inhibition performance. A comparison of the OD values among different samples reveals that the pristine titanium foil exhibits the highest OD value. After the growth of 1D TiO_2_ on the titanium foil via anodic oxidation, the OD value decreases to a certain extent. When different amounts of MoS_2_ are loaded, the OD value of the composites first decreases and then increases. Specifically, the MoS_2_/TiO_2_-1 composite possesses the smallest OD value, demonstrating the optimal photocatalytic antibacterial inhibition effect. This result is consistent with the antibacterial performance order determined by the plate colony counting method, further confirming the reliability of the experimental findings.

### 2.3. Photoelectrochemical Properties

**Figure 7:**
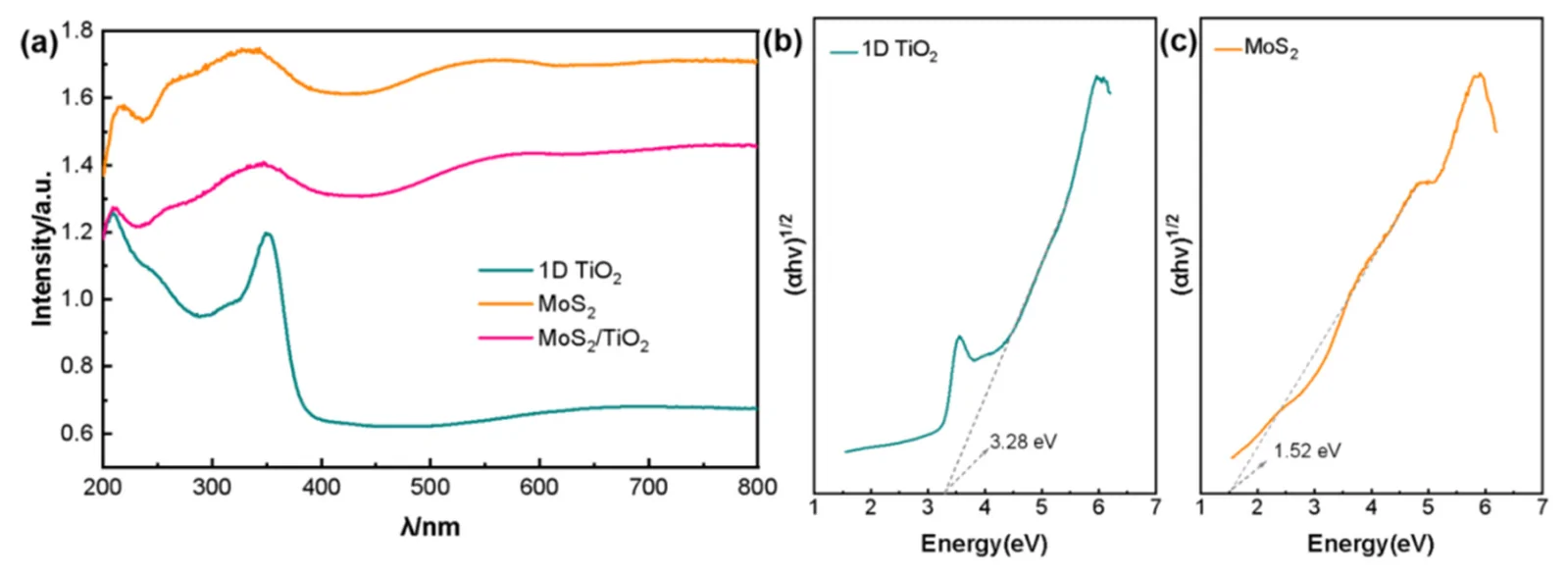
UV–visible diffuse reflectance spectra (DRS) of TiO_2_, MoS_2_, and MoS_2_/TiO_2_ (**a**); plots of (Ahν)^1/2^ vs. photon energy (hν) of TiO_2_ (**b**) and MoS_2_ (**c**). The dashed arrows indicate the tangent lines used to extrapolate the optical band gap energy.

Ultraviolet–visible (UV–vis) absorption spectroscopy was employed to investigate the optical absorption properties of 1D TiO_2_, pristine MoS_2_, and MoS_2_/TiO_2_ (MoS_2_/TiO_2_-1 selected as a representative sample). As shown in Figure 7a, the light absorption range of 1D TiO_2_ is mainly confined to the ultraviolet (UV) region. After loading MoS_2_ onto the 1D TiO_2_ surface, the absorption range of MoS_2_/TiO_2_ is significantly extended into the visible region, indicating a remarkable enhancement in visible-light absorption (up to ~600 nm). As illustrated in Figure 7b,c, the band gap energy (E_g) of the samples was calculated from the UV–vis absorption spectra. According to the literature [41], the band gap energy of 1D TiO_2_ is 3.28 eV. For MoS_2_, the band gap energy was determined to be 1.52 eV, consistent with reported values [42,43].

**Figure 8:**
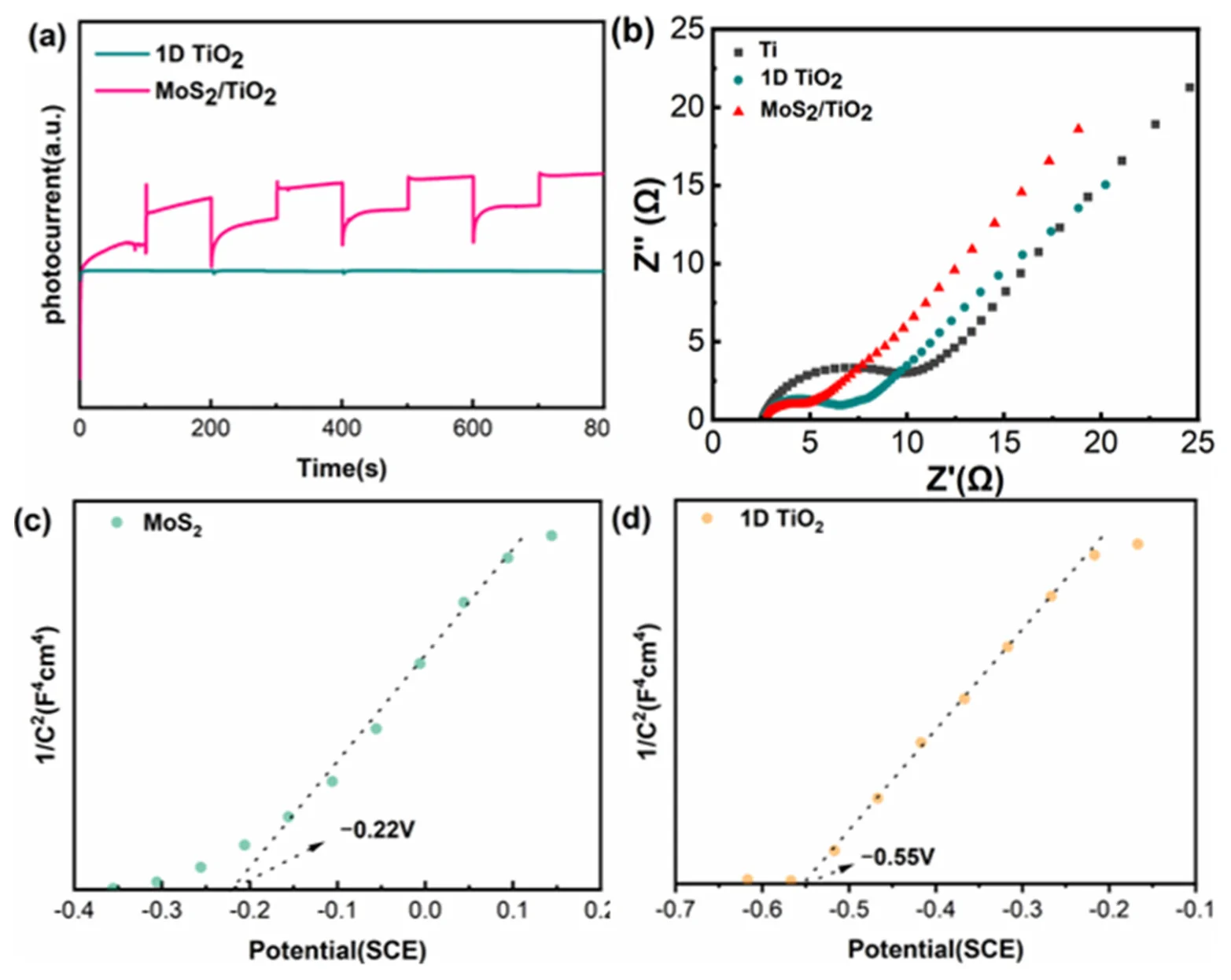
Photocurrent density–time curves of TiO_2_ and MoS_2_/TiO_2_ (**a**); electrochemical impedance spectra of titanium foil, 1D TiO_2_, and MoS_2_/TiO_2_ (**b**); Mott–Schottky plots of MoS_2_ (**c**) and 1D TiO_2_ (**d**). The dashed arrows indicate the intercepts of the fitted lines on the potential axis, corresponding to the flat-band potential.

Photocurrent response and electrochemical impedance spectroscopy (EIS) were measured to explore the charge separation behavior of 1D TiO_2_ and MoS_2_/TiO_2_ (Figure 8). Transient photocurrent response measurements were utilized to evaluate the photocarrier separation efficiency of the as-prepared samples, which serves as a critical descriptor of photocatalytic performance [44]. Upon illumination with a xenon lamp, the photocurrent of the MoS_2_/TiO_2_ composite rapidly increased to its maximum value. Immediately after the light was switched off, the photocurrent promptly decayed back to the baseline, indicative of a fast and reversible photoresponse behavior (Figure 8a). This favorable characteristic arises from the efficient separation and suppressed recombination of photogenerated electrons and holes within the composite. Notably, the photocurrent intensity remained nearly unchanged over three consecutive on–off cycles, verifying the outstanding photostability and operational durability of the sample. Furthermore, the photocurrent intensity of the MoS_2_/TiO_2_ composite was considerably higher than that of pristine 1D TiO_2_, mainly attributed to the formation of a heterojunction at the MoS_2_/TiO_2_ interface induced by MoS_2_ coupling, which effectively promotes the separation and interfacial transfer of photogenerated charge carriers, lowers their recombination rate, and consequently strengthens the photocurrent response.

Variations in the semicircle radius of EIS spectra reflect the photogenerated electron–hole separation performance, with a smaller radius corresponding to superior charge-separation efficiency [37]. Compared with the unanodized Ti foil, 1D TiO_2_ exhibits a smaller EIS semicircle radius; furthermore, the MoS_2_/TiO_2_ composite shows an even smaller semicircle radius than 1D TiO_2_ (Figure 8b). This phenomenon indicates that the in situ loading of an appropriate amount of MoS_2_ can facilitate the separation of photogenerated electrons and holes, thereby effectively promoting electron transfer [45].

Mott–Schottky measurements were performed to further analyze the interfacial charge-transfer characteristics. Both 1D TiO_2_ and MoS_2_ were identified as n-type semiconductors from the positive slopes of the Mott–Schottky plots (Figure 8c,d). With a saturated calomel electrode (SCE) as the reference electrode, the flat-band potentials of MoS_2_ and 1D TiO_2_ were determined to be −0.22 V and −0.55 V, respectively. Converted to the normal hydrogen electrode (NHE) scale via E(NHE) = E(SCE) + 0.241 V, the corresponding flat-band potentials are +0.02 V and −0.31 V, respectively. Since the flat-band potential is typically 0.1–0.3 V more positive than the conduction band (CB) edge for n-type semiconductors [46], the CB potentials of MoS_2_ and 1D TiO_2_ are estimated to be −0.18 V and −0.51 V, respectively. Combined with the band-gap energies (E_g) determined from UV–vis diffuse reflectance spectroscopy (E_g,TiO_2_ = 3.28 eV; E_g,MoS_2_ = 1.52 eV, (Figure 7b,c), the valence band (VB) positions of the two semiconductors were further derived to be +2.77 V (TiO_2_) and +1.34 V (MoS_2_) vs. NHE.

### 2.4. Photocatalytic Mechanism

The XPS valence band spectra of TiO_2_ and MoS_2_ were analyzed (Figure 3f). The position of the valence band edge relative to the Fermi level can be obtained from the valence band spectrum [39], indicating that the valence band maximum (VBM) of TiO_2_ and MoS_2_ lies ~2.68 eV and ~1.19 eV below the Fermi level, respectively (corresponding to VBM of +2.77 V and +1.34 V vs. NHE, with Fermi levels at +0.09 V and +0.15 V vs. NHE). The detailed band structure parameters of all samples are summarized in Table 1.

**Figure 9:**
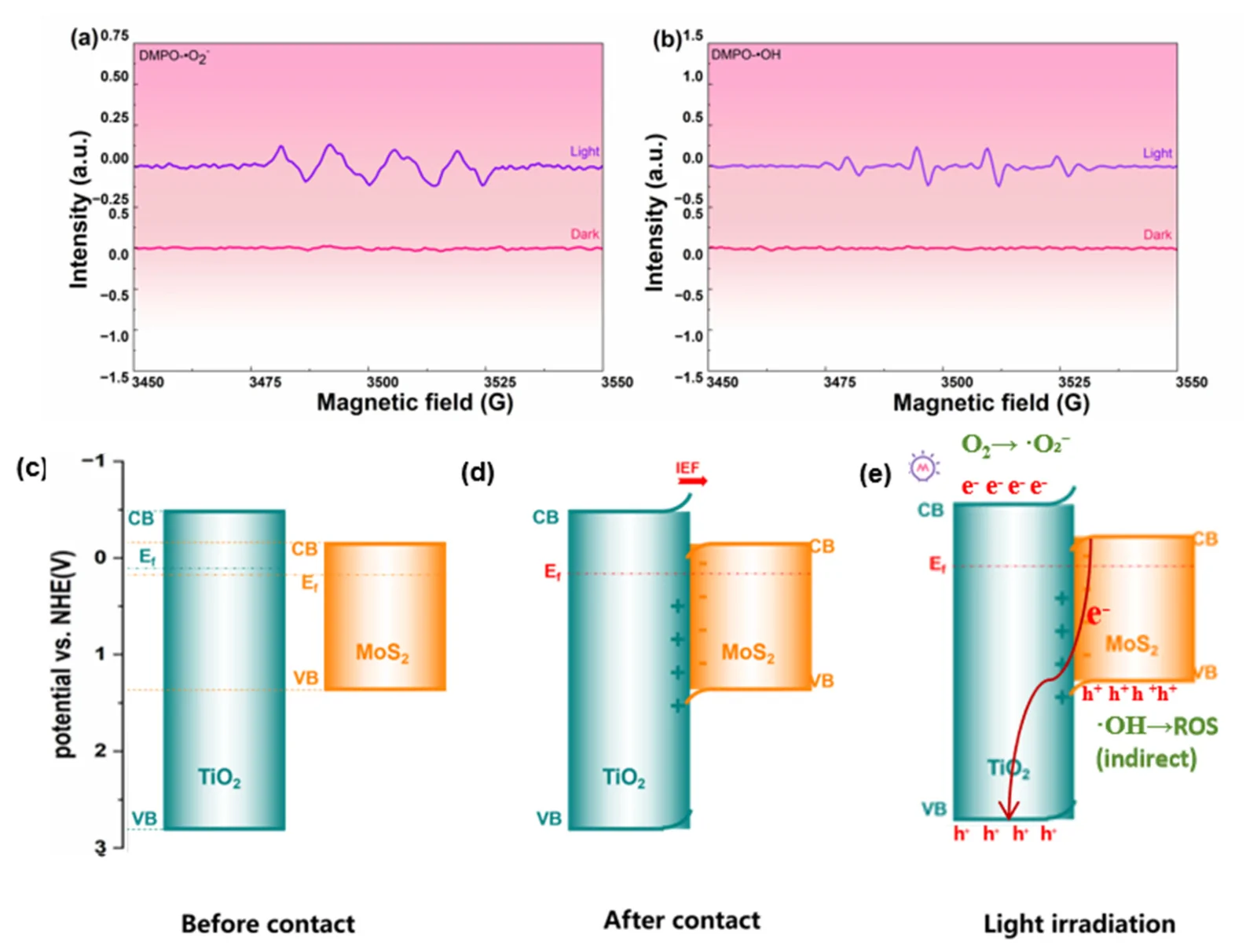
EPR spectra of DMPO-•O_2_^−^ (**a**) and DMPO-•OH (**b**) recorded under light irradiation; schematic illustration of the promoted photoelectron transfer from the MoS_2_ conduction band to the TiO_2_ valence band (S-scheme interfacial recombination under the internal electric field): before contact (**c**), after contact (**d**), and under light irradiation (**e**). The red arrows show the migration pathways of photogenerated charge carriers; the IEF arrow denotes the direction of the built-in electric field.

To further explore the photocatalytic antibacterial mechanism of MoS_2_/TiO_2_ composites, electron paramagnetic resonance (EPR) spectroscopy was employed to identify reactive free radicals generated during photocatalytic reactions. The 5,5-dimethyl-1-pyrroline N-oxide (DMPO) spin trap was utilized to detect superoxide radicals (•O_2_^−^) and hydroxyl radicals (•OH). As shown in Figure 9a,b, no characteristic DMPO-•O_2_^−^ or DMPO-•OH adduct signals were detected under dark conditions. By contrast, distinct and strong characteristic signals of the two radical adducts were clearly observed for the MoS_2_/TiO_2_ composite under full-spectrum simulated solar light (300 W Xe lamp, no optical filter). We note that no chemical radical-scavenging experiments were performed in this study; instead, the strict light/dark contrast and the concurrent detection of both •O_2_^−^ and •OH directly establish the photocatalytic origin of the reactive oxygen species.

Combined with the relative band positions and radical trapping experimental results, we propose a plausible photocatalytic antibacterial mechanism of MoS_2_/TiO_2_ (Figure 9c–e). Since TiO_2_ has a higher Fermi level than MoS_2_, electrons on the TiO_2_ surface spontaneously migrate to MoS_2_ upon contact between the two materials. This process induces upward band bending of TiO_2_ and downward band bending of MoS_2_, accompanied by the formation of a built-in electric field directed from TiO_2_ to MoS_2_, which is the driving force that stabilizes the S-scheme charge flow [47]. Upon light absorption, valence-band electrons of the MoS_2_/TiO_2_ composite are continuously excited to the conduction band, while an equal number of holes is simultaneously generated in the valence band. Driven by the built-in electric field and the interfacial band bending, the less-negative conduction-band electrons of MoS_2_ (−0.18 V vs. NHE) migrate toward the TiO_2_ side and selectively recombine with the valence-band holes of TiO_2_ at the interface. This S-scheme recombination preserves the strongly reducing conduction-band electrons of TiO_2_ (−0.51 V vs. NHE) and the spatially separated valence-band holes of MoS_2_, while suppressing non-selective migration of the other charge carriers. The fabricated MoS_2_/TiO_2_ forms an S-scheme heterojunction structure, which retains the strong redox capability of the junction and achieves efficient carrier separation via the built-in electric field, thereby facilitating the generation of ROS.

The assignment of an S-scheme (rather than Type-II) charge-transfer pathway is supported by a combination of mutually consistent observations: (i) the concurrent EPR detection of •O_2_^−^ and •OH under illumination, a profile consistent with a heterojunction that preserves the strongly reducing electron pool of the TiO_2_ CB together with the spatially separated holes of the MoS_2_ VB, whereas a Type-II transfer would accumulate the surviving electrons on the less-negative MoS_2_ CB (−0.18 V) and thermodynamically prevent O_2_ → •O_2_^−^; (ii) the retention of the reducing TiO_2_ CB (−0.51 V vs. NHE, sufficient for O_2_ → •O_2_^−^ at −0.33 V vs. NHE); and (iii) the ≈45-fold photocurrent enhancement, consistent with efficient, spatially separated charge migration. Direct interfacial probes, such as HRTEM of the MoS_2_/TiO_2_ interface and work-function measurements, would provide complementary microstructural confirmation and are planned for further work.

It is widely acknowledged that ROS play a crucial role in cell death. During the antibacterial process, ROS attack the cell membrane as the primary contact site, inducing severe intracellular damage, including lipid peroxidation, protein oxidation, and DNA damage [48,49,50]. The interconversion between various ROS facilitates this process. Such an S-scheme heterojunction enables efficient separation of photogenerated electron–hole pairs while retaining strong photocatalytic reduction activity. The absence of radical signals in the dark and their clear appearance under illumination demonstrate that •O_2_^−^ and •OH are generated exclusively by the photocatalytic process, confirming that the antibacterial activity arises from light-driven ROS generation.

As summarized in Tables S1–S3, the MoS_2_/TiO_2_ composite delivers a full-spectrum bactericidal efficiency of 86% against *Staphylococcus aureus* (at a 1:1000 dilution), clearly outperforming the bare 1D TiO_2_ control (33%, corresponding to a reduction in colony count from 597 to 398 in Figure 6a) under identical conditions. It likewise surpasses visible-light TiO_2_-based systems (e.g., Au/Pt-modified TiO_2_ nanotubes, <50% [51]). Although absolute efficiencies ≈100% have been reported, these generally rely on more favorable conditions—full-spectrum + photothermal synergy in powder/film form, enzymatic/photocatalytic nanoflowers, or upconversion doping [22,29,52]—whereas the present self-supported architecture retains full activity without a recovery step, which is advantageous for practical disinfection. Consistent with the mechanistic evidence above, the enhanced charge separation (≈45-fold photocurrent) and the band structure summarized in Table 1 support the dominant •O_2_^−^/•OH-mediated antibacterial mechanism, as further corroborated by EPR. In interpreting these results, we note that the MoS_2_ valence-band edge (+1.34 V vs. NHE) is not positive enough to oxidize water or surface hydroxyl groups directly, so the detected •OH is more plausibly formed indirectly through reduction of dissolved oxygen by the retained TiO_2_ conduction-band electrons, i.e., via a •O_2_^−^ → H_2_O_2_ → •OH cascade, rather than by direct hole oxidation. Because no chemical radical-scavenging or quantitative spin-trapping experiments were performed, the present EPR data establish the photocatalytic generation and identity of •O_2_^−^ and •OH but do not quantify their individual contributions; the ROS-based conclusions are therefore limited to this qualitative mechanistic interpretation.

## 3. Materials and Methods

Photocatalysts were synthesized using the following reagents: ammonium fluoride (NH_4_F) was obtained from Sinopharm Group Chemical Reagent Co., Ltd. (Shanghai, China); absolute methanol (CH_3_OH), ethanediol ((CH_2(_OH)_2_), and ethanol (C_2_H_6_O) were purchased from Tianjin Obokai Chemical Co., Ltd. (Tianjin, China); ammonium heptamolybdate ((NH_4_)_6_Mo_7_O_24_) was supplied by Tianjin Fengboat Chemical Reagent Technology Co., Ltd. (Tianjin, China); sodium hydroxide (NaOH) was acquired from Tianjin Best Chemical Co., Ltd. (Tianjin, China); and thiourea (CH_4_N_2_S) and anhydrous sodium sulfate (Na_2_SO_4_) were provided by Shanghai Aladdin Biochemical Technology Co., Ltd. (Shanghai, China).

### 3.1. Fabrication of MoS_2_/TiO_2_

Preparation of TiO_2_. One-dimensional (1D) TiO_2_ was fabricated via a two-step anodization method. Titanium foil (99.7% purity, 0.1 mm thickness) was obtained from Baoji Ruicheng Titanium Metal Co., Ltd. (Baoji, Shaanxi, China). The titanium foil served as the anode, and a platinum sheet was used as the cathode. The titanium foil was cut into 1.0 cm × 1.0 cm pieces and ultrasonically cleaned (KQ2200, Ningbo Xinyi Ultrasonic Equipment Co., Ltd., Ningbo, China) sequentially in acetone, methanol, and deionized water for 15 min to remove surface contaminants. The electrolyte consisted of 0.5 wt% NH_4_F and 2.5 vol% deionized water in anhydrous ethylene glycol and was magnetically stirred (RCT B S-025, Thermo Fisher Scientific (China) Co., Ltd., Shanghai, China) for 60 min at room temperature before anodization. First, a pre-anodization step was conducted at 40 V for 2 h at room temperature using a direct-current regulated power supply (Maynuo M8813, Nanjing Meirnuo Electronics Co., Ltd., Nanjing, China). The treated titanium foil was then ultrasonically rinsed with deionized water to completely remove the as-formed oxide layer, thereby exposing a pre-patterned (dimpled) surface. Subsequently, the secondary anodization was performed under the same conditions (40 V, 2 h, room temperature) to grow a hierarchical 1D TiO_2_ architecture composed of a nanotube bottom layer whose surface nanowires extend and interconnect into groove structures; the use of identical parameters in the two steps is consistent with established two-step anodization protocols [53,54]. Finally, the obtained 1D TiO_2_ samples were annealed in a muffle furnace (KSL-1200X, Hefei Kejing Materials Technology Co., Ltd., Hefei, China) at 500 °C for 2 h with a heating rate of 2 °C/min.

Preparation of MoS_2_/TiO_2_ Composites. In this work, the MoS_2_/TiO_2_ composites were prepared by the in situ growth of MoS_2_ on the surface of the prefabricated TiO_2_ via a hydrothermal method. Different amounts of the Mo source—ammonium heptamolybdate, (NH_4_)_6_Mo_7_O_24_ (0.5, 1, 2, and 3 mmol)—and the corresponding amounts of the S source—thiourea, CH_4_N_2_S—were weighed on an electronic analytical balance (FA2204B, Shanghai Yueping Scientific Instrument Co., Ltd., Shanghai, China) and dissolved in 30 mL of deionized water at a S:Mo molar ratio of 4:1. After ultrasonic dispersion, the homogeneous solution was transferred into a hydrothermal reactor, and the pre-synthesized 1D TiO_2_ samples were immersed into the solution. The hydrothermal reaction was conducted at 200 °C for 24 h under precise temperature control. After the reaction, the reactor was naturally cooled to room temperature, and the obtained samples were collected, repeatedly rinsed with deionized water and absolute ethanol to remove any surface residues, and finally dried in an oven. A series of MoS_2_/TiO_2_ composites with different MoS_2_ loading amounts was successfully obtained and named MoS_2_/TiO_2_-0.5, MoS_2_/TiO_2_-1, MoS_2_/TiO_2_-2, and MoS_2_/TiO_2_-3 according to their MoS_2_ content.

### 3.2. Characterization

The crystal structure of the samples was characterized via X-ray diffraction (XRD; D/MAX2200, Rigaku Co., Tokyo, Japan). The surface morphology and elemental distribution were observed using a field-emission scanning electron microscope (FE-SEM; JSM-7900F, JEOL, Tokyo, Japan). X-ray photoelectron spectroscopy (XPS; ESCALAB 250, Thermo Fisher Scientific, Waltham, MA, USA) was employed to analyze the chemical composition and elemental valence states of the prepared samples. The optical absorption properties were measured by UV–visible diffuse reflectance spectroscopy (DRS; Cary 5000, Agilent, Santa Clara, CA, USA). Photoelectrochemical tests, including electrochemical impedance spectroscopy (EIS), transient photocurrent response (I–t), and Mott–Schottky (MS) measurements, were performed on an electrochemical workstation (CHI-760E, Chenhua Instrument Co., Ltd., Shanghai, China). All spectral, diffraction, and electrochemical data were processed and plotted using Origin (OriginLab Corporation, Northampton, MA, USA).

### 3.3. Photoelectrochemical Tests

Photoelectrochemical (PEC) measurements. The photoelectrochemical properties of the as-prepared samples were evaluated using a standard three-electrode system. The as-prepared sample served as the working electrode, a saturated calomel electrode (SCE) as the reference electrode, and a platinum sheet as the counter electrode. A 0.5 mol/L Na_2_SO_4_ aqueous solution was used as the electrolyte to ensure stable electrochemical responses. Transient photocurrent (I–t), electrochemical impedance spectroscopy (EIS), and Mott–Schottky (M–S) measurements were performed using a CHI-760E electrochemical workstation. 

For the I–t measurements, the working electrode was illuminated with a 300 W Xe lamp (CEL-PF300-T10, China Education Au-light Co., Ltd., Fengtai, Beijing, China) providing full-spectrum simulated solar light (no optical filter). The light intensity at the electrode surface was measured to be 100 mW/cm^2^ using a radiometer (CEL-NP2000, China Education Au-light Co., Ltd., Beijing, China). This light source is consistent with the full-spectrum (simulated-solar) illumination conditions used in the photocatalytic antibacterial experiments.

The exposed (apparent) working electrode area used for photocurrent density normalization was 1.0 cm × 1.0 cm (1.0 cm^2^), matching the monolithic sample. All photocurrent densities were calculated with respect to this exposed area.

### 3.4. Photocatalytic Antibacterial Experiment

Liquid Luria–Bertani (LB) medium was prepared by dissolving 1 g of tryptone, 0.5 g of yeast extract, and 1 g of NaCl in 100 mL of deionized water. The flask was capped with aluminum foil and autoclaved (SQ810C, Chongqing Yamato Technology Co., Ltd., Chongqing, China) at 121 °C for 20 min, followed by natural cooling prior to use. For solid LB medium, 2 g of tryptone, 1 g of yeast extract, 2 g of NaCl, and approximately 3 g of agar powder were suspended in 200 mL of deionized water, the pH was adjusted to 7.0 ± 0.2 using a 5 mol/L NaOH solution, and the mixture was autoclaved at 121 °C for 20 min with foil caps. After the medium had cooled to approximately 50 °C, 15–20 mL aliquots were aseptically poured into sterile Petri dishes. Pipette tips were autoclaved at 121 °C under 103.4 kPa (15 psi) for 2 h and subsequently dried at 60 °C for 4 h.

The frozen bacterial strain (*Staphylococcus aureus*, ATCC 51650) was thawed at room temperature for 15 min under aseptic conditions in a clean bench (CJT, Suzhou Jinjing Purification Equipment Technology Co., Ltd., Suzhou, China), and the surface of the cryovial was disinfected with cotton swabs saturated with 75% ethanol. An aliquot of the bacterial suspension was aseptically transferred into pre-warmed LB broth using a sterile pipette, and the culture was incubated at 37 °C with orbital shaking at 180 rpm for 24 h in a shaking incubator (ZWY-2102C, Shanghai Zhicheng Analytical Instrument Manufacturing Co., Ltd., Songjiang, Shanghai, China).

Prior to sample treatment, the cultured bacterial solution was diluted with sterile LB medium and grown to the exponential phase: 70 μL of the original bacterial suspension was mixed with 35 mL of sterile LB medium and incubated in a constant-temperature incubator (SHP-250, Shanghai Jinghong Laboratory Equipment Co., Ltd., Shanghai, China) at 37 °C for 12 h.

Antibacterial activity test:The antibacterial activity was evaluated by optical density (OD) measurement and the plate-counting method under full-spectrum simulated-solar illumination, with a dark-cultured group used as the control to exclude the contribution of dark adsorption. Three groups were tested under identical conditions to assess the influence of MoS_2_ loading on the antibacterial performance of TiO_2_: (i) bare titanium (Ti), (ii) anodized TiO_2_ (the hierarchical 1D nanotube–nanowire architecture), and (iii) the MoS_2_/TiO_2_ composite photocatalyst.

Briefly, 20 μL of the activated bacterial suspension was added to 20 mL of LB liquid medium in a centrifuge tube and mixed thoroughly, standardizing the initial bacterial load by a 1:1000 dilution; three parallel groups were prepared. Each as-prepared sample (a monolithic sheet of 1.0 cm × 1.0 cm) was separately immersed in the bacterial suspension (20 mL) and cultured in a shaking incubator (ZWY-2102C, Shanghai Zhicheng Analytical Instrument Manufacturing Co., Ltd., Songjiang, Shanghai, China) at 37 °C under full-spectrum simulated-solar illumination from a 300 W Xe lamp (CEL-PF300-T10, China Education Au-light Co., Ltd., Fengtai, Beijing, China; no optical filter) for 6 h at a light intensity of 100 mW/cm^2^ (measured with a CEL-NP2000 radiometer), while the control group was cultured under identical conditions in the dark.

After incubation, the solid sample was removed from the culture, and 200 μL of the light-incubated suspension was transferred into a sterilized 96-well plate; the OD_600_ value was recorded using a microplate reader (Thermo-G20, Thermo Fisher Scientific (China) Co., Ltd., Shanghai, China) at 600 nm. For plate counting, 20 μL of the light-incubated suspension was spread uniformly onto LB solid medium with a spreader (three parallel plates per sample); the plates were inverted and incubated at 37 °C in a constant-temperature incubator (SHP-250, Shanghai Jinghong Laboratory Equipment Co., Ltd., Shanghai, China) for 24 h to promote optimal colony growth, after which the colonies were counted and photographed. All measurements were performed in triplicate (*n* = 3). The bactericidal efficiency was calculated from the viable cell counts of the treated group relative to the dark control.

### 3.5. EPR Measurement

Electron paramagnetic resonance (EPR) spectra were recorded at room temperature on a Bruker Elexsys-II E 500 spectrometer (X-band; Bruker BioSpin GmbH, Rheinstetten, Germany). 5,5-Dimethyl-1-pyrroline N-oxide (DMPO, ≥97%, Sigma-Aldrich, St. Louis, MO, USA) was used as the spin-trapping reagent at a final concentration of 100 mM. For each measurement, a monolithic piece of the composite (1.0 cm × 1.0 cm) was cut into 2 mm × 2 mm pieces, and all pieces were immersed in 1.0 mL of the corresponding solvent (deionized water for hydroxyl-radical detection and methanol for superoxide-radical detection). For the detection of hydroxyl radicals (•OH), the photocatalyst was dispersed in water, whereas for the detection of superoxide anions (•O_2_^−^), the photocatalyst was dispersed in methanol. The suspension was kept in the dark or illuminated with a 300 W Xe lamp (CEL-PF300-T10, China Education Au-light Co., Ltd., Fengtai, Beijing, China) providing full-spectrum simulated solar light (no optical filter). Dark controls were recorded under identical conditions without illumination, and no radical signals were observed in the dark for either species.

## 4. Conclusions

In summary, a novel S-scheme MoS_2_/TiO_2_ heterojunction composite was successfully fabricated for photocatalytic antibacterial applications. One-dimensional (1D) TiO_2_ nanostructures were grown on titanium foil via a secondary anodization method. The introduction of TiO_2_ nanowires created abundant groove structures on the TiO_2_ nanotube surface, providing sufficient active sites for the uniform loading of MoS_2_ nanosheets. UV–vis spectroscopy and electrochemical measurements verified the construction of efficient electron transport channels between the MoS_2_ and TiO_2_ interfaces. Under light irradiation, the built-in electric field drives the selective interfacial recombination of the electrons at the conduction band minimum (CBM) of MoS_2_ with the holes at the valence band maximum (VBM) of TiO_2_, while the strongly reducing electrons at the TiO_2_ CBM and the holes at the MoS_2_ VBM are retained at the respective redox-active sites. This charge separation mechanism preserves the strong reduction capability at the TiO_2_ CB and efficient interfacial charge separation, promotes the generation of ROS (•O_2_^−^ and •OH), and ultimately enhances the photocatalytic antibacterial performance. This work provides a useful framework for the further development and optimization of high-efficiency photocatalytic antibacterial materials.

## Figures and Tables

**Table 1:** Flat-band-derived band parameters of 1D TiO_2_ and MoS_2_ (all potentials are given versus NHE).

Samples	1D TiO_2_	MoS_2_
conduction band CB (vs. NHE) (V)	−0.51	−0.18
band gap E_g (eV)	3.28	1.52
valence band VB (vs. NHE) (V)	2.77	1.34
Fermi level *E*_f_ (vs. NHE) (V)	0.09	0.15
VB-*E*_f_ (eV)	2.68	1.19

## Data Availability

The original contributions presented in this study are included in the article/Supplementary Material. Further inquiries can be directed to the corresponding author.

## Supplementary Materials

The following supporting information can be downloaded at https://www.mdpi.com/article/10.3390/ijms27219763/s1.
