# Supplementary material for: Construction of S-Scheme Heterojunction MoS2/TiO2 and Investigation of the Photocatalytic Inhibition Performance
Source: Int J Mol Sci. 2026 Oct 30;27(21):9763. doi: 10.3390/ijms27219763 (PMC13762837; doi:10.3390/ijms27219763)
Supplement: Supplementary file 1 [file ijms-27-09763-s001.zip › ijms-4556073-supplementary.pdf]

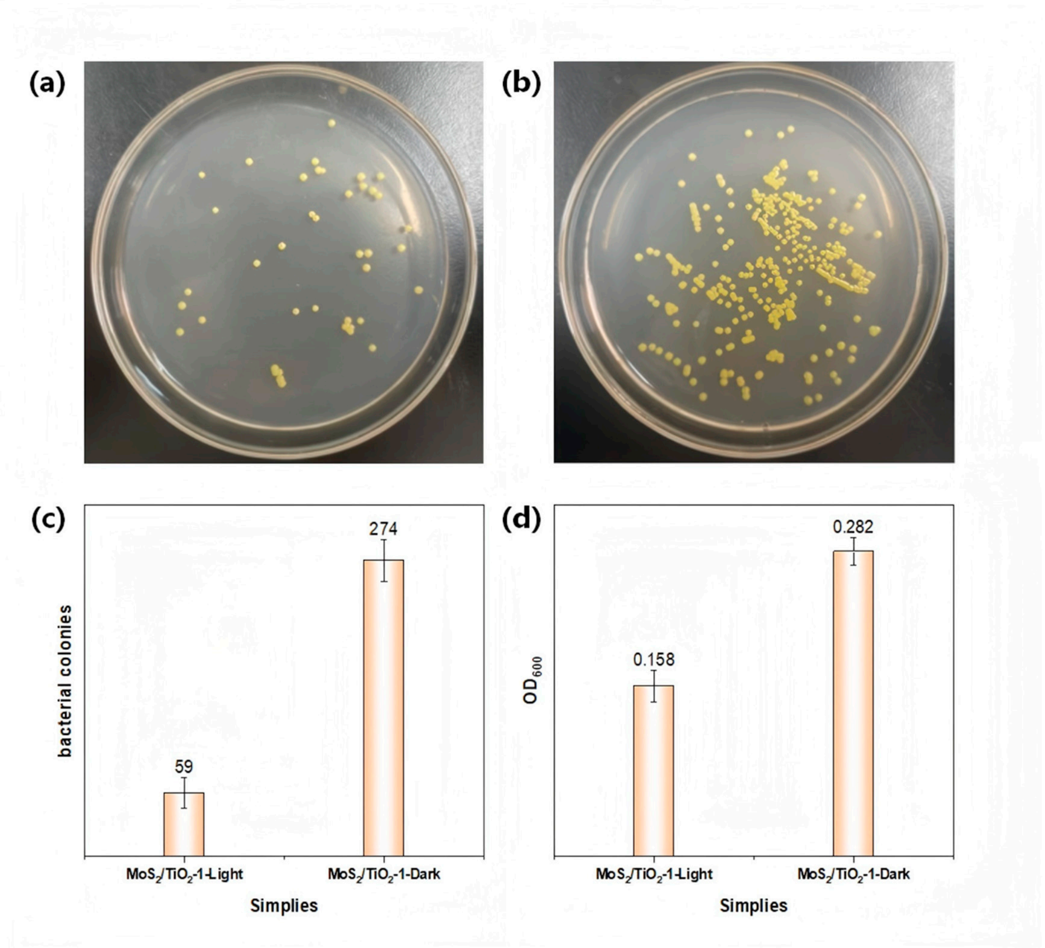

Figure S1. Photocatalytic antibacterial experiments of MoS_2/TiO_2against Staphylococcus aureus under dark and light irradiation. Images under dark conditions(a);Images under light irradiation (b) ; the Staphylococcus aureus colony number (c) and OD value(d) of MoS2/TiO2-1 under dark and light irradiation.

Supplementary Table S1: Quantitative comparison in the PEC / band-structure (photocurrent + band) dimension

System	Heterojunction type	Photocurrent density / PEC response	Band-structure parameters (vs NHE)	Ref.
This work: 1D MoS_2/TiO_2 (self-supported)	S-scheme	Steady-state net photocurrent ≈0.12 mA/cm²; pristine TiO_2 ≈0.0026 mA/cm²; ≈45×	TiO_2: CB −0.51 / VB +2.77 V (Eg ≈ 3.2 eV); MoS_2: CB −0.18 / VB +1.34 V (Eg ≈ 1.3–1.5 eV); $\cdot O_2^-/\cdot OH$ detected by EPR	This work
2D/1D MoS_2/TiO_2 heterostructure	S-scheme (simulated solar) ↔ Type-II (visible-light) switchable	Transient photocurrent markedly enhanced (MoS_2/TiO_2 outperforms bare MoS_2 and TiO_2)	Fermi-level alignment → built-in field MoS_2→TiO_2; S-scheme charge transfer under simulated solar light (DFT-supported)	[S1]
MoS_2/TiO_2 nanotube arrays (TNAs)	Type-II	210.6 μA/cm² (= 0.21 mA/cm²) @ 1.23 V vs RHE, ≈18.2× pure TNAs (11.5 μA/cm²)	MoS_2 bandgap ≈ 1.9 eV extends TiO_2 visible-light absorption	[S2]
1T-MoS_2@TiO_2 nanotubes	Type-II / Schottky	Enhanced photocurrent + reduced EIS arc radius	TiO_2: CB −0.4 / VB +2.8 V; 1T-MoS_2: CB +0.18 / VB +1.98 V (Eg 3.17 → 2.84 eV)	[S3]

Supplementary Table S2. Comparison of the photocatalytic antibacterial performance of MoS_2/TiO_2-based and related TiO_2-based photocatalysts

**System (structure)**	**Light source**	**Initial [bacteria]**	**Bactericidal efficiency (%)**	**Notes on driving conditions**	**Ref.**
**This work: 1D TiO_2/MoS_2 (self-supported, S-scheme)**	300 W Xe, full spectrum, 100 mW/cm^2	1:1000	**86**	Full-spectrum; recovery-free	This work
**This work: pristine 1D TiO_2 (control)**	300 W Xe, full spectrum, 100 mW/cm^2	10^5 CFU/mL	**36**	Full-spectrum	This work
BC/MoS_2/TiO_2 (bacterial cellulose film)	Sunlight (UV+Vis+NIR, PTT+PDT)	—	99.9929 (*S. aureus*) / 99.8935 (*E. coli*)	Full-spectrum + photothermal, fine powder/film	[S4]
TiO_2 NTs @Au / @Pt	Visible (470 nm)	10^5 CFU/mL	Au <50 / Pt <40	Same load as this work	[S5]
Ce,Er co-doped TiO_2 (0.5Ce0.2Ti–O)	Simulated sunlight, 20 min	10^7 CFU/mL	91.23 (*S. aureus*) / 92.8 (*E. coli*); bare TiO_2 44.85	doped, NIR-conversion	[S6]
TiO_2 NTs@MoS_2 nanoflowers	Visible	—	strong vs. ESBL *E. coli* & MRSA	wound-healing setting	[S7]

Supplementary Table S3: Comparison of Antibacterial Activity of MoS_2/TiO_2Composites with Different Architectures (Structure – Activity Dimension)

Composite architecture	Preparation method	Light source	Strain	Antibacterial effect	Ref.
1D TiO_2/MoS_2 (this work, self-supported)	Anodization + hydrothermal	Full-spectrum (300 W Xe, 100 mW/cm²)	S. aureus	86%	This work
TiO_2 NTs@MoS_2 nanoflowers (core–shell loading)	Anodization + hydrothermal	Visible light	ESBL E. coli, MRSA	Strong (incl. photothermal, wound-healing)	[S7]
BC/MoS_2/TiO_2 flexible composite film	In situ biosynthesis (bacterial cellulose)	Sunlight (UV+Vis+NIR; PTT+PDT)	S. aureus / E. coli	99.9929 / 99.8935%	[S4]
2D/1D (nanosheet-on-nanorod) MoS_2/TiO_2	Hydrothermal	Solar/visible light	—	PEC enhancement (with photocurrent)	[S3]

Note: The 2D/1D (nanosheet-on-nanorod) MoS_2/TiO_2row is included to illustrate the charge-separation/PEC advantage of the 2D/1D architecture; the cited work is a photoelectrochemical – photocatalytic study rather than an antibacterial report, so no antibacterial strain or inhibition data is listed.

Reference

S1. A. Hezam, K. Alkanad, M. A. Bajiri, J. Strunk, K. Takahashi, Q. A. Drmosh, N. Al-Zaqri, L. N. Krishnappagowda, 2D/1D MoS_2/TiO2 Heterostructure Photocatalyst with a Switchable CO_2 Reduction Product. *Small Methods* **2023**, 7, 2201103, doi: org/10.1002/smtd.202201103.

S2 1.Nguyen, P.H.; Cao, T.M.; Nguyen, T.T.; Tong, H.D.; Van Pham, V. Photoelectrochemical water oxidation over TiO_2 nanotubes modified with MoS_2 and g-C_3N_4. *Beilstein J Nanotechnol* **2022**, *13*, 1541-1550, doi:10.3762/bjnano.13.127.

S3. Chen, M.G.; Sun, T.; Zhao, W.; Yang, X.R.; Chang, W.Y.; Qian, X.X.; Yang, Q.; Chen, Z. In Situ Growth of Metallic 1T-MoS_2 on TiO_2 Nanotubes with Improved Photocatalytic Performance. *Acs Omega* **2021**, *6*, 12787-12793, doi:10.1021/acsomega.1c01068.

S4. Liu, S.; Lou, H.; Luo, J.; Albashir, D.; Shi, Y.; Chen, Q. A Novel In Situ Biosynthesized Bacterial Cellulose/MoS_2/TiO_2 Composite Film for Efficient Removal of Dyes and Pathogenic Bacteria from Industrial Wastewater under Sunlight Illumination. *ACS Appl. Mater. Interfaces* **2025**, *17* (13), 19543–19561. DOI: 10.1021/acsami.4c21052.

S5. Moon, K.S.; Park, Y.B.; Bae, J.M.; Choi, E.J.; Oh, S.H. Visible Light-Mediated Sustainable Antibacterial Activity and Osteogenic Functionality of Au and Pt Multi-Coated TiO_2 Nanotubes. *Materials (Basel)* **2021**, *14*, doi:10.3390/ma14205976.

S6. Ren, Y.; Han, Y.; Li, Z.; Liu, X.; Zhu, S.; Liang, Y.; Yeung, K. W. K.; Wu, S. Ce and Er Co-doped TiO_2 for rapid bacteria-killing using visible light. *Bioactive Materials* **2020**, *5* (2), 201–209, doi: 10.1016/j.bioactmat.2020.01.001.

S7. Lin, Y.; Liu, X.; et al. Visible-Light-Driven Photocatalysis-Enhanced Nanozyme of TiO_2 Nanotubes@MoS_2 Nanoflowers for Efficient Wound Healing Infected with Multidrug-Resistant Bacteria. *Small* **2021**, *17*, 2103348. Erratum in *Small*. **2021**, 17(39):e2103348. doi: 10.1002/smll.202103348.
